# Hospital contextual factors affecting the implementation of health technologies: a systematic review

**DOI:** 10.1186/s12913-021-06423-2

**Published:** 2021-05-01

**Authors:** Adriano Grossi, Ilda Hoxhaj, Irene Gabutti, Maria Lucia Specchia, Americo Cicchetti, Stefania Boccia, Chiara de Waure

**Affiliations:** 1 Section of Hygiene, University Department of Life Sciences and Public Health, Università Cattolica del Sacro Cuore, Largo Francesco Vito, 1, 00168 Rome, Italy; 2 Graduate School of Health Economics and Management (ALTEMS), Faculty of Economics, Università Cattolica del Sacro Cuore, Rome, Italy; 3 Clinical Governance Unit, Fondazione Policlinico Universitario A. Gemelli IRCCS, Rome, Italy; 4 Department of Woman and Child Health and Public Health - Public Health Area, Fondazione Policlinico Universitario A. Gemelli IRCCS, Rome, Italy; 5 Department of Medicine and Surgery, University of Perugia, Perugia, Italy

**Keywords:** Technology, Implementation, Hospital, Systematic review, Health technology assessment

## Abstract

**Background:**

To keep a high quality of assistance it is important for hospitals to invest in health technologies (HTs) that have the potential of improving health outcomes. Even though guidance exists on how HTs should be introduced, used and dismissed, there is a surprising gap in literature concerning the awareness of hospitals in the actual utilization of HTs.

**Methods:**

We performed a systematic literature review of qualitative and quantitative studies aimed at investigating hospital contextual factors that influence the actual utilization of HTs. PubMed, Scopus, Web of Science, Econlit and Ovid Medline electronic databases were searched to retrieve articles published in English and Italian from January 2000 to January 2019. The quality of the included articles was assessed using the Critical Appraisal Skills Programme checklist for qualitative studies, Newcastle-Ottawa Scale for the cross-sectional studies and the Mixed Methods Appraisal Tool for mixed method studies.

**Results:**

We included 33 articles, which were of moderate to high methodological quality. The included articles mostly addressed the contextual factors that impact the implementation of information and communication technologies (ICTs). Overall, for all HTs, the hospital contextual factors were part of four categories: hospital infrastructure, human resource management, financial resources and leadership styles.

**Conclusion:**

Our systematic review reported that the contextual factors influencing the HTs utilization at hospital level are mainly explored for ICTs. Several factors should be considered when planning the implementation of a new HTs at hospital level. A potential publication bias might be present in our work, since we included articles published only in English and Italian Language, from January 2000 to January 2019. There remains a gap in the literature on the facilitators and barriers influencing the implementation and concrete utilization of medical and surgical HTs, suggesting the need for further studies for a better understanding.

**Supplementary Information:**

The online version contains supplementary material available at 10.1186/s12913-021-06423-2.

## Introduction

Globally, healthcare systems are facing great challenges due to increasing ageing population, chronic diseases, and economic restraints. Hospitals are under an increasing pressure to provide high quality services in an era of limited financial resources and growing expectations from patients and society [1]. Even though health technologies (HTs) play a key role and guidance exist on how these should be introduced, used and dismissed from hospitals [2–8], there is a gap in literature concerning the “awareness” of hospitals in the utilization of HTs. The relevance of the context has been reported in a study conducted among 53 hospital managers from nine European countries [9]. Although there are several attempts to identify contextual factors through different methodological approaches, such as expert panels and interviews, literature reviews, or research, there is not a clear consensus on how to define or assess them [10, 11]. Contextual factors have been defined as “the set of characteristics and circumstances or unique factors that surround a particular implementation effort” [12]. However, it is not clear whether it is the contextual factors that are affected by HTs, or if they may also affect the HTs’ use in the first place. Among the most relevant attempts to theorize the dynamics existing between these dimensions, there is Leavitt’s diamond framework, which describes the relationship between organizational structure, tasks, people and technology as well as the implications for change [13]. Furthermore, the relevance of hospitals’ structural dimensions has been previosuly described in studies investigating the contextual factors that influence the adoption of HTs [14, 15]. Nevertheless, the available evidence is still far from sufficient to draw clear conclusions on the impact that contextual dimensions have on the effective utilization of HTs and their value to patients’ care. Indeed, most of the studies have focused on the process of formal adoption of HTs rather than on their concrete utilization and effective integration [14–16]. This poses great challenges to research efforts since these processes are deeply different one from the other, and are affected by organizational, individual, environmental, and innovation-related determinants [17, 18]. These determinants should be considered in order to create a more cost effective, resource efficient, informed health care service. In fact, the factors underlying successful implementations are not only those leading to the acquisition and introduction of a new technology, but also those capable of creating conditions that favor the effective and safe use of the new technology. The latter aspect is much less investigated. In addition, previous studies focusing on organizational or managerial factors affecting HTs’ implementation, have not considered their collocation within a specific organizational setting, generating confusion between characteristics that may be ascribable to hospitals rather than to primary care settings [16, 19]. Moreover, previous systematic reviews addressed these issues focusing on a specific HT, instead of considering all HTs’ common factors [19]. These contextual factors, considered as essential to successful implementation of healthcare innovations [20], are barely recorded, analysed or considered when implementing change [21]. There is a clear need to investigate the impact of contextual factors on the actual use of HTs and on the value they bring to hospitals and patients. Such evidence is essential considering the changes in hospitals’ organizational models and managerial policies and could lead to re-interpretations of Health Technology Assessment (HTA) reports, in light of a hospital’s organizational scenario, overcoming the risk of rather uncritical applications, regardless of the specific context. Therefore, we conducted a systematic review to identify and summarize the contextual factors, in terms of hospital-specific organizational and managerial factors, that can affect the concrete implementation of HTs.

## Methods

The systematic review is reported according to the Preferred Reporting Items for Systematic Reviews and Meta-Analyses (PRISMA) guidelines [22] (Additional file 1). The protocol of this systematic review has been included in the internal report of the Improved methods and actionable tools for enhancing HTA (IMPACT_HTA) Project (data not publicly available).

### Search strategy

Data sources used were PubMed, Scopus, Web of Science, Ovid Medline and EconLit. Each electronic database was searched to identify relevant articles published in English and Italian language form January 1st, 2000 to January 1st, 2019. We have chosen this time range considering the fact that HTs have evolved a lot and quickly, therefore we think that it would not be appropriate or useful to compare the technological set-up of 30 years ago with that of today. We developed a search string consisting of the search terms and keywords. The keywords “hospital”, “secondary care”, “tertiary care”, “biomedical technology”, “technology”, “facilitator”, “barrier”, “management”, “organizational”, “adoption, “acceptance”, “implementation”, “assimilation”, “uptake”, “utilization”, were combined using Boolean Operators and Medical Heading Subjects terms. The search strategy used in all the databases is reported in Additional file 2.

### Eligibility criteria

The search question and the eligibility criteria for inclusion in the systematic review were developed according to the PICOS framework.

- P (population) - secondary or tertiary hospitals located within the European Union (EU) or within the Organisation for Economic Co-operation and Development (OECD);
- I (intervention) - HT, using the definition of the World Health Organization (WHO) for HTs, as “the application of organized knowledge and skills in the form of devices, medicines, vaccines, procedures and systems developed to solve a health problem and improve quality of lives” [23, 18].;
- C (comparator) - There is not a comparator applicable for the studies in this systematic review;
- O (outcome) - Contextual factors that facilitate or inhibit the implementation of a HT in a hospital setting;

We referred to those factors related to organization, infrastructure, support and capacity, team structure and collaboration, motivation to change, leadership and resources, that could potentially mediate the effect of a HT implementation and use.

- S (study type) - Quantitative and qualitative studies reporting primary data were included.

Considering the PICOS developed for this systematic review, studies assessing contextual factors, facilitators and barriers related to the implementation of a HT in a secondary or tertiary hospital were considered eligible. Due to a lack of a standardised definition of implementation, we searched for all the concepts related to it, such as “adoption”, “implementation”, “acceptation” and “assimilation”. Therefore, we included the articles that evaluated the actual utilization of a given HT, referring to it as “HT implementation”.

Studies that evaluated the technology implementation in primary care settings or the mere introduction of a HT in a hospital were excluded. Reviews (narrative, scoping or systematic), editorials, commentaries, conference abstracts and theoretical studies were excluded.

### Study selection

The identified articles from all the databases were uploaded to Mendeley Software and the duplicates were removed. Two researchers (AG; IH) independently performed the initial step of screening based on title and abstracts. In a second step, two independent researchers (AG; IH) read carefully the articles with full texts available in order to decide the final articles to be included in the systematic review. Articles satisfying the eligibility criteria were selected for inclusion in the systematic review. Reasons for exclusion of full texts were recorded and were reported in the PRISMA flow chart. The reference lists of the included articles were carefully hand-searched to retrieve additional eligible articles. Any discrepancies in the screening process and study selection were resolved by consulting a third reviewer (IG).

### Data extraction and synthesis

Two researchers (AG; IH) independently extracted from each article the following data:

1. Study identification (first author, year of publication);
2. Study characteristics (country, study design, population, study size, response rate (in case of survey);
3. Information related to the HTs investigated (HT type, and the contextual factors affecting their implementation).

Any discrepancies in the data extraction process were resolved through discussion with a third author (IG).

Afterwards, we labelled the extracted contextual factors according to their topic and added up the studies that displayed each one. Additionally, these factors were grouped into the four areas: hospital’s financial resources, leadership styles, human resource management and hospital infrastructure. Within each area, the contextual factors were described as either impeding or facilitating the implementation of the HT, as reported by each study. Each factor was checked in duplicate by two researchers (IH, AG.) and incongruence were resolved through discussion. If a study performed statistical analysis in order to investigate the relation between a given factor and the HT implementation, variables were categorised as facilitator in case of positive association and as barrier in case of negative association.

We performed a synthetic descriptive analysis of the studies according to the area of contextual factors.

### Quality assessment

To assess the quality of the included studies we used the Critical Appraisal Skills Programme (CASP) checklist for qualitative studies [24]. As for the cross-sectional studies, the adapted version of Newcastle-Ottawa Scale (NOS) for cross-sectional studies was used [25]. The Mixed Methods Appraisal Tool [26] was used to assess the quality of mixed method studies. Two independent researchers (AG, IH) evaluated each article, and any disagreements were resolved through discussion.

## Results

### Bibliographical search

**Fig. 1:**
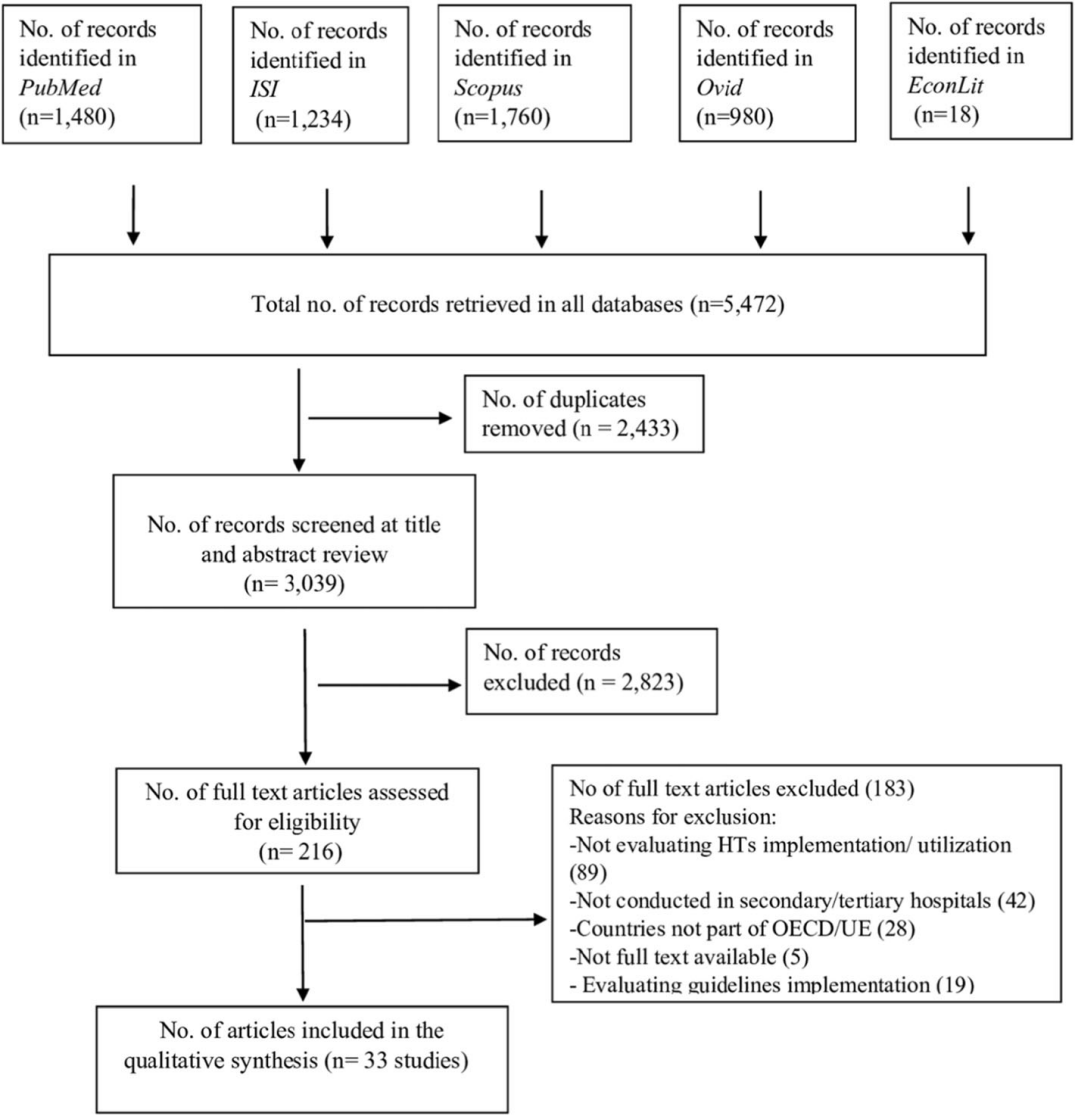
Flow chart of literature search strategy

The search strategy identified 5472 articles from the five databases, as showed in detail in Fig. 1. After removing the duplicates, 3039 articles were retained for titles and abstract screening. A total of 216 full texts articled were read. After the screening process, 33 studies, [27–59] reporting original primary data, met the eligibility criteria for this systematic review.

### Description of the studies

The main characteristics of the included studies are reported in Table 1. Twenty-seven (81.8%) articles [27–36, 38–40, 45–48, 50–59] reported multicenter studies, involving up to 4606 hospitals. Seventeen studies (54.6%) [27, 31, 32, 39, 41, 42, 44–46, 49, 50, 52, 53, 56–59] used qualitative approaches such as focus group discussions or descriptive methods, 13 studies (39.3%) [28, 33–36, 38, 40, 43, 47, 48, 51, 54, 55] applied a quantitative study design through surveys, while three studies (9%) [29, 30, 37] implemented mixed methods. The response rate, when reported, varied from 12.5% [33] to 78% [37]. The recruited participants were mainly hospital key stakeholders, hospitals’ executives, physicians, nurses and clinical information officers (CIOs). Of the 33 studies, 12 (36%) were conducted in USA [29, 30, 34, 37, 38, 44, 47–49, 51, 53, 58], five (15%) in Canada [28, 39, 40, 50, 52] and three (9%) [31, 35, 41] in Australia. Among European countries, the majority of studies were conducted in UK [42, 45, 46, 56, 57] and Germany [33, 55, 59], five (15%) and three (9%) studies, respectively, followed by other countries with only one study. As for HTs investigated, 28 studies (82%) evaluated information technologies (ITs), such as: electronic health record (EHR) [31, 36–38, 40, 42, 47, 48, 54, 56, 57], computerized physician order entry (CPOE) [53], health information technology (HIT) [28, 33, 44, 51, 58], standardized inpatient discharge model (SDM) [50], picture archiving and communication system (PACS) [39, 43], computerized decision support systems (CDSS) [27, 46]. Three studies (9%) analyzed the following surgical interventions: thrombolysis procedure [52], transcatheter aortic valve implementation (TAVI) [59] and minimally invasive cardiac surgery (MICS) [30], and two studies (6%) evaluated organizational technologies such as a surgical perioperative checklist for risk management [52] and palliative care in dementia and cancer settings [55].

**Table 1:** Characteristics of the thirty-three included studies in the systematic review

First Author	Year	Country	Study Design	Technology	Study setting	Study size	Study Population
Bomba D	2006	Australia	qualitative	EPDS	one public hospital	26	medical staff, pharmacist, nurses, clinical IT experts
Cuccinello M	2015	UK	qualitative	EMR	one teaching hospital in central Scotland	19	CIOs, directors, clinicians, nurses
Debono D	2017	Australia	qualitative	EMR	2 teaching hospitals	19	nurses
Dharampl N	2016	Canada	qualitative	Surgical perioperative safety checklist	3 acute care hospitals in Calgary	31	surgeons, anesthesiologists, nurses
Duyck P	2010	Belgium	survey	PACS	one teaching hospital	362	physicians, radiologists
Edmondson AC	2003	USA	mixed method	Minimally invasive cardiac surgery (MICS)	150 hospitals	165	surgeons, anesthesiologist, nurses
Gosling A	2003	Australia	survey	HIT	3 hospitals	180	clinicians
Granlien MF	2008	Denmark	survey	EMR	all hospitals in Region Zealand	232	physicians, nurses, managers
Hao H	2011	USA	survey	PDA	2 hospitals in Pennsylvania	138	physicians
Hubner U	2010	Austria; Germany	survey	HIT	270 hospitals in Germany and 45 in Austria	45; 270	270 hospitals in Germany, 45 hospitals in Austria
Kazley AS	2007	USA	survey	EMR	4606 hospitals	4606	all nonfederal, US general and surgical acute care hospitals
Merkel S	2015	Germany	qualitative	TAVI	9 hospitals	10	cardiologists, cardiosurgeons
Moeckli J	2013	USA	mixed method	Tele-Intensive care unit (ICU)	7 hospitals	97	ICUs staff
Nakamura MM	2013	USA	survey	EHR	126 children’s hospitals	93	CIOs
Nanji KC	2009	USA	qualitative	pharmacy bar code scanning system	one tertiary care center in Boston	150	physicians, nurses
Olson JR	2012	USA	survey	HIT	104 hospitals in Minnesota	210	CEOs, CMO
Paré G	2010	Canada	qualitative	HIT	acute care hospitals in Québec and Ontario	106	CIOs
Paré G	2007	Canada	qualitative	PACS	2 hospitals	454	physicians
Poon EG	2004	USA	qualitative	CPOE	26 hospitals	52	physicians
Randell R	2010	UK	qualitative	CDSS	3 general hospitals	74	physicians, nurses, managers
Scholten N	2015	Germany	survey	Systemic thrombolysis treatment	all hospitals that treated ischemic stroke patients	286	neurology departments
Sheikh A	2011	UK	qualitative	EHR	12 hospitals in England	431	healthcare professionals, managers, administrative staff
Shen X	2012	USA	survey	EHR	21 oncology department facilities	21	CIOs, clinicians
Sommerbakk R	2016	Norway	qualitative	Palliative care tool	2hospitals	20	physicians, nurses, CIOs
Struik MH	2014	Netherlands	survey	EMR	several hospitals (exact number not available)	298	physicians, nurses
Szydlowski S	2009	USA	qualitaitve	HIT	one local hospital in Pennsylvanya	6	CIOs, nurses
Takian A	2014	UK	qualitative	EHR	2 hospitals	48	CIOs
Tuot DS	2015	USA	qualitative	HIT	22 hospitals	16	CIOs
Urowitz S	2008	Canada	survey	EHR	83 general and acute care hospitals	83	CEOs, CIOs
Vadillo PC	2016	USA	mixed methods	EHR	one small urban hospital	71	therapists, nurses
Varonen H	2008	Finland	qualitative	CDSS	hospitals in seven areas of Finland	39	physicians
Woiceshyn J	2017	Canada	qualitative	Standardized inpatient discharged model (SDM)	5 urban hospitals	39	hospital excecutives, frontline managers
Xie Y	2016	UK	qualitative	ICT	2 NHS Hospital Trusts	51	healthcare professionals in oncology and cardiology department

*Abbreviations: HIT* Health information technology, *EHR* electronic health record, *PDA* personal digital assistant, *EMR* electronic health records, *PACS* picture archiving and communication system, *PDA* personal digital assistant, *CDSS* computerized decision support systems, *CPOE* computerized physician order entry, *TAVI* transcatheter aortic valve implementation, *EPDS* electronic prescribing decision support, *ICT* Information communication technology, *CEO* Chief Executive Officers, *CMO* Chief Medical Officer, *CIO* Chief Information Officers

### Quality assessment

Seventeen studies (51.5%) [27, 31, 32, 39, 41, 42, 44–46, 49, 50, 52, 53, 56–59], employing qualitative methods, were assessed using CASP (Additional file 3). The qualitative methods used were identified in all the studies, namely focus groups or semi-structured interviews. There was a clear statement of the objectives and a rational explanation why the qualitative study design was the most appropriate method for the research aim. Among all the studies, the researchers had explained in detail the selection criteria [56]. Ethical dimensions were reported in 12 studies (70.5%) [27, 31, 32, 41, 42, 49, 50, 52, 56–59], in which institutional ethics committee approval and informed consent from participants were obtained. Data collection strategy was pertinent for specific qualitative method, and data analysis, sufficiently rigorous in all the studies, included a general coding of the interview transcripts based on the constructs of the conceptual framework used. In most of the studies, the qualitative analysis of the interview transcripts was conducted with an inductive approach, grouping the coded transcript text and then tabulating the items whereas, in three of them (17.7%) [32, 46, 59] a software was used. In all the studies, the authors pointed out the study limitations and the contribution of the results to the existing knowledge. The discussion of the findings was supported by evidence and directions for future research were suggested.

Thirteen studies (39%) [28, 33–36, 38, 40, 43, 47, 48, 51, 54, 55], using quantitative methods, were appraised by NOS for cross-sectional studies (Additional file 4). In all the studies, the authors had described the sampling strategy, which mostly was representative of the target population. Not all the studies had compared the characteristics of the respondents and non-respondents while over half of them had reported the response rate. In two studies (15%) [38, 40], with an unsatisfactory response rate, the authors called for caution in interpreting the findings. The questionnaire used was not previously validated in five studies (38%) [34, 36, 43, 54, 55], in which this was explicated in detail in the respective methods section. In all the studies, the most important confounding factors were controlled, and the statistical tests used for data analysis were clearly explained. Eleven studies (85%) [28–30, 33–36, 38, 40, 43, 48, 51, 54] used questionnaires for the outcome ascertainment. After evaluating if the specific criteria were met in each study, the score ranged from five to eight, appraising studies of moderate to high quality.

The Mixed Methods Appraisal Tool was used to assess the quality of three studies [29, 30, 37] that had clearly demonstrated why the mixed method was chosen to address the research question (Additional file 5). The criteria of each method were satisfied by all the studies. Only in two studies [29, 30] the qualitative and quantitative components were effectively integrated to answer the research question and the outputs were adequately interpreted. Vadillo et colleagues [37] reported different outcomes interpretations for the two components, focus group and semi-structured interviews.

### Evidence from the studies

The contextual factors relevant for the implementation of HTs are reported in Table 2 and in details in Additional file 6. These factors can be ascribable to four main areas: hospital’s financial resources, leadership styles, human resource management and hospital infrastructure.

**Table 2:** Summary of determinants influencing the actual utilization of HTs

Areas of determinants	Influence of determinants on actual utilization of HTs	References
**Financial support and adequate budgeting**	Hospital financial resources hinders actual utilization	[22–27, 35, 36, 39–41, 45, 48, 54, 55]
	High cost technologies force the hospital to consider other priorities	[48]
	Financial difficulties impact on recruiting and retaining technical staff	[41, 48]
	Reimbursement policies might facilitate HT utilization	[53, 54]
	Financial self-sufficiency represents a necessary condition	[34]
	Funding model for specialist clinician reimbursement facilitates HT utilization	[53]
**Leadership**	Leadership enables to:	
	-shorten the gap between implementation team and end users	[52]
	-overcome resistance to change	[48]
	-ameliorate understanding of HT and tasks	[27]
	-involve end users in the decision-making process	[24, 27, 34, 37, 44, 49]
	-positively influence attitude toward new HTs	[29, 34, 44, 53]
	-adapt the HT integration to the needs of the work environment	[34, 41]
	Champions and top opinion leaders drive the integration	[45]
	Champions facilitate communication and team work	[54, 25]
**Human Resources Management**	Human Resources Management enables to:	
	- define and resource new roles capable of supporting and sustaining the change	[34, 37, 41, 44, 54]
	- appropriately manage staff recruitment preventing staff shortages and contractual tensions	[26, 27, 31, 33, 34, 38, 39, 48, 51]
	- ensure appropriate training to end users	[24, 32, 33, 37, 38, 38, 41, 44, 49, 51, 52]
	- take into account concerns about time-consuming training	[39, 49, 36]
	- address concerns about new HT (e.g. changes to workload, workflow) - improve cooperation and team working	[22, 26, 31, 36, 44, 47, 51, 34, 44, 48, 53, 54]
**Structure**	Technological capability is a latent process that enables HT utilization	[23, 28, 33, 34, 36, 42, 43, 46, 50–52, 54]
	Large size and urban location might positively influence utilization	[42, 23]
	Teaching hospitals might be positively associated with utilization	[43]

The first area concerned hospital’s financial resources. According to the results of 13 studies (39%) [27–29, 32, 40, 41, 44–46, 50, 53, 59, 60], financial support and adequate budgeting are of utmost importance for successful HTs implementation. Resources have been identified as a barrier when hospitals faced difficulties obtaining funding for high-cost technology or for recruiting technical staff. Poon et al. [53] stated that the high cost of CPOE implementation forced hospital officials to consider other priorities. The absence of a budget that is coherent with organizational units’ technological assets may hinder the possibility of their concrete use. Merkel et al. [59], while investigating the use of TAVI among cardiologists and cardio-surgeons, reported cost issues and lack of reimbursement policies as the main cause of a sub-optimal use of the HT.

Second, a total of 12 articles (36%) [28, 30, 32, 34, 42, 44, 50, 53, 57–59] mentioned leadership as a required factor to successfully implement HTs. In most of these studies, researchers found that a persistent and sustained leadership by top management was a key element to successfully implement the HT. Takian et al. [57] stated that an insightful leadership, as well as the managerial team which was signed-up to the vision of EHR, were crucial for its implementation, acting as a boundary spanner that bridged the gap between implementation team and end users. Moreover, Poon et al. [53] considered leadership as one of the major strategies to overcome resistance to change that inevitably occurred in CPOE implementation among physicians. In a study conducted in Norway [32], researchers demonstrated that distant management led to a lack of detailed understanding of the tasks involved in patient care by operators. Further, the fact of nurses not being represented in the management group, and therefore not involved in the decision-making processes regarding organizational change, was a possible barrier to the implementation of HTs. Factors such as managers’ attitude and propensity to involve staff members in decision-making processes were considered a facilitator factor by six studies (18.1%) [29, 32, 39, 42, 49, 54]. In addition, opinion leaders seemed to influence peers’ attitudes towards a new technology [34, 39, 49, 58]. The opinion leader’s effect is strictly connected, although not coincident, to top management’s support and leadership style. Frontline managers of urban hospitals in Canada, as the executive champions, tend to integrate innovation initiatives with ongoing projects, so that staff incorporation would be as supportive as possible [50]. In the same line, in Merkel et al. [59] and in Edmondson et al. [30], the presence of a champion was essential in facilitating the dialogue between the cardiologists and cardiosurgeons, in order to drive the implementation forward. Managing integration into current practice was also held to be fundamental. Often HTs led to the reorganization of departments and workflows [49, 52, 53, 58]. As a consequence, some authors stated that the implementation strategy should take several aspects into account and adapt specifically to the working environment needs rather than simply being a “technical” project [39, 46].

Third, Human Resource Management (HRM) was another important factor emerging in several studies. HRM appears to cover a crucial role in the phase of staff supply and planning, due to the need to define new roles, including those in charge to support the change [39, 42, 46, 49, 59]. Insufficient or inadequate human resources, staff shortages, lack of staff recruitment and contractual tensions were considered barriers for HTs implementation in nine (26%) studies [31, 32, 36, 38, 39, 43, 44, 53, 56]. Moreover, HRM is also essential to plan education and to inform providers. To effectively use a given technology it is often necessary to undergo an appropriate training program and many studies underlined that the inability to satisfy true training needs was considered a major barrier for implementation [29, 37, 38, 42–44, 46, 49, 54, 56, 57]. Although it was stated that education might be a time-consuming activity [44, 54] and should be cautiously planned [41], in general training programs are considered among the most determinant factors for an effective implementation. Experts and clinicians in Bomba et al. [41], concerned about the feasibility of training initiatives in a 24-h, 7-days per week operating environment, proposed the application of innovative methods of training such as video and online courses. Nevertheless, not only training in a strict sense but also “general communication” may play a major role. Indeed, some authors noticed that the utilization of a HT could cause a perceived waste of time or an extra workload, inducing utilizers to “reject” the HT or to use it against their will, in sub-optimal ways [27, 31, 36, 41, 49, 52, 56]. In this context, it is important that HRM tools are coherent with the hospital organizational and technological asset [24] and spur operators to “accept” the HT, addressing their concerns, including those related to workflow changes [39, 49, 53, 58, 59]. That was the case of the staff acceptance of a telemedicine ICU where participants complained the HT was disrupting staff communication making the job harder [24]. A final dimension in which HRM was held to make a solid difference is the generation and allocation of specific behavioral competencies. In particular, team-working emerged as a fundamental factor for HTs’ implementation in 14 studies (42%) [29–32, 35, 39, 44, 49, 50, 52, 53, 57, 59]. For instance, the study by Edmonson et al. [30], conducted with 16 multi-disciplinary teams of medical staff in 16 hospitals, suggested that team communication led to the successful implementation of surgical procedures. Further, according to their results, teams with better internal communication are likely to expand also external communication with other teams, empowering the implementation process. In other studies, researchers found that a lack of mutual communication and team cooperation is a limiting factor for HTs' implementation [31, 52, 59].

Finally, evidence also suggests that some determinants related to the infrastructure of the hospitals might influence HTs’ implementation. For instance, the technological capability of the hospital (the characteristics of the set of all other technologies available) or even the affiliation to some universities or to a multihospital network, could be associated to HTs' implementation [28, 33, 38, 39, 41, 47, 48, 51, 55–57, 59] .

## Discussion

The present work provides a novel point of view in understanding how hospital contextual factors may hinder or facilitate a full implementation of HTs. Although HTA is attentive to capture the effects of technologies on multiple domains (among which the organizational one), the inverse relationship, i.e. how organizational/contextual factors may affect HTs’ implementation, remains unexplored. Addressing this query implies ascribing to HTs the power of “producing value” within patients’ continuum of care. The term “value”, here, is ascribable to Porter’s definition, which is defined as “the patient health outcomes achieved per dollar spent”. As stated by Porter, “value encompasses many of the other goals already embraced in healthcare, such as quality, safety, patient-centeredness, and cost containment, and integrates them. It is also fundamental to achieving other important goals such as improving equity and expanding access at reasonable cost” [61].

This systematic review provides valuable insights concerning the relationship between contextual factors and HTs’ implementation. Our results, in line with previous evidence, showed that facilitators and barriers to HTs' implementation vary across studies and countries. However, this work suggests that facilitators and barriers to the actual utilization of HTs could be reconducted to four overarching domains. The first dimension concerns the hospital’s availability of financial resources. Although intuitive in principle, this finding has further implications than the mere ascertainment that a hospital with more resources is advantaged in implementing the use of HTs. Indeed, implications concern the internal distribution of resources and their coherence with organizational units’ technological assets. This, in turn, provides food for thought on the consequences of misaligning HTs and budgets within or across organizational units.

The second dimension that affects HTs’ implementation refers to leadership styles and management. What seems to emerge here is that HTs should be driven in a very participative manner. Top management involvement is required to implement HTs, suggesting that an excessive decentralization of responsibilities to lower levels may hinder implementation. On the other hand, it emerges clearly that top management has a role of “mediation” of professionals, to be exerted by involving professionals at all levels - including nurses and final utilizers of the HT. This provides interesting implications in terms of managerial styles, suggesting an increased effectiveness of attitudes that approach a concern for people rather than a concern for results [62].

Third, HRM appears fundamental in utilizing HTs. HRM seems to have a major impact in terms of planning people’s work, both through “standard” training pathways and the development of behavioral skills such as, team-working. Development and integration of technical and behavioral skills is challenging, especially in a dynamic and complex sector such as healthcare, where the need for education is of utmost importance. In particular, future research should address the relevant topic of how incentive schemes may affect the use of HTs. This may be of great interest in an era in which hospitals are increasingly required to be accountable and tend to introduce Management by Objectives logics within their daily functioning [63]. Finally, no evidence was identified in terms of managerial accounting tools. It is expected that a structured, integrated, and well-functioning set of tools, able to collect and analyze data, would affect the use of HTs. This is strictly related to hospitals’ ability and swiftness in enhancing communication within and across hospital units, as well as at the inter-organizational level.

The role of of the identified contextual factors could depend on the environmental context in which the hospital operates. A systematic review that evaluated the influence of context on quality improvement reported that variations in the characteristics of the external context in different sites, such as physical environment, socio-cultural context or political and funding environment can influence implementation outcome [64]. Future research should examine whether some specific contextual factors are more important in specific settings, to provide the information needed to translate the technology implementation to different settings and situations.

Although this systematic review employed a robust methodological approach and it was rigorously conducted, few limits need to be identified. In the first place, as in all systematic reviews, our study might be subject to publication bias. Even though the search strategy was performed in five databases, we only included articles published in English and Italian language, from January 1st, 2000 until January 1st, 2019, suggesting that we may have missed a number of articles. We acknowledge that the search strategy is rather extensive in scope and may lack analytical evidence on specific aspects of HTs' implementation. Even though the search may not have identified all relevant literature, this risk of publication bias has been minimised by manually screening the reference lists of the included articles. However, although it is of utmost importance to analytically target specific features of implementation, with attention to specific types of HTs, the intent of the authors was to generate a broad evidence of the state of the art in literature, in order to assess the desirable directions of future research. We also acknowledge that, even if we restricted our search to OECD, differences across countries (e.g. private or public healthcare systems) might be relevant and generalization of our findings should be cautious. Even though our systematic review was not restricted to any type of HTs, the vast majority of studies concerned information and communication technologies, and nothing emerged in reference to clinical equipment and drugs.

Understanding the relationship occurring between hospital contextual factors and the implementation of HTs is an arduous task. On one hand it is clear that HTs may affect the context, on the other the inverse relationship also seems to hold true. Although HTA has definitively incorporated the organizational domain within its span of interest, the way in which this domain may enable or hinder a full implementation of HTs is still rather unclear. This work has the potential to contribute to a better understanding in this direction. Policy makers often face challenges when making decisions regarding the evaluation and implementation of HTs. Therefore, we believe that our results might contribute to the development of strategies addressing these factors, aiming a successful implementation.

## Conclusions

To conclude, there seem to exist two main gaps in literature: one concerning the contextual factors of interest that could play a role when implementing a HT. Although relevant evidence has emerged, several items, such as managerial accounting tools may have been overlooked. The second, −and perhaps more salient one, concerning the typologies of HTs investigated. Elucidating contextual factors is essential to identify effective, sustainable, and reproducible strategies that aim to overcome the barriers and improve HTs' implementation. Future research is needed to shed light in this direction, providing guidance to hospital management, to reduce the uncertainty of the concrete effects produced by HT in settings that present numerous contingent factors.

## Supplementary Information

**Additional file 1.**

**Additional file 2.**

**Additional file 3.**

**Additional file 4.**

**Additional file 5.**

**Additional file 6.**

## Data Availability

The datasets used and/or analysed during the current study are available from the corresponding author on reasonable request.

## Abbreviations

HIT: Health information technology
EHR: Electronic health record
PDA: Personal digital assistant
EMR: Electronic health records
PACS: Picture archiving and communication system
PDA: Personal digital assistant
CDSS: Computerized decision support systems
CPOE: Computerized physician order entry
TAVI: Transcatheter aortic valve implementation
EPDS: Electronic prescribing decision support
ICT: Information communication technology
CEO: Chief Executive Officers
CMO: Chief Medical Officer
CIO: Chief Information Officers
PRISMA: Preferred Reporting Items for Systematic Reviews and Meta-Analyses
