# Supplementary material for: Hospital contextual factors affecting the implementation of health technologies: a systematic review
Source: BMC Health Serv Res. 2021 May 1;21:407. doi: 10.1186/s12913-021-06423-2 (PMC8088675; doi:10.1186/s12913-021-06423-2)
Supplement: Supplementary file 2 — Additional file 2. [file 12913_2021_6423_MOESM2_ESM.docx]

Title: Hospital contextual factors affecting the implementation of health technologies: A systematic review

**Search Strategy**

**PubMed**

(("Hospitals"[Mesh] OR "Secondary Care"[Mesh] OR "Tertiary Healthcare"[Mesh]) AND ("Diffusion of Innovation"[Mesh] OR "Biomedical Technology"[Mesh] OR "medical technology" OR "health technology" OR "health innovation" OR "medical innovation" OR "innovative technology" OR "Technology"[Mesh] OR "medical device" OR "new drug") AND (("facilitator" OR "enabler" OR "barrier" OR "inhibitor" OR management OR "Organization and Administration"[Mesh]) AND (adopt* OR accept* OR uptake OR implement* OR assessment OR utilization OR assimilat* OR measurement)))**Filters:**Journal Article, English, Italian, from 2000/1/1 - 2019/1/1

**Scopus**

(("secondary care"  OR  "tertiary care"  OR  hospital)  AND  (( innovative  W/2  technology )  OR  "biomedical technology" )  AND  ( ( organizational  OR  management  OR  barrier  OR  facilitator  OR  enabler )  AND  ( adoption  OR  implementation  OR  acceptance  OR  evaluation  OR  assessment  OR  measurement  OR  utilization  OR  assimilation ) ) )  AND  ( LIMIT-TO ( SRCTYPE ,  "j" ) )  AND  ( EXCLUDE ( AFFILCOUNTRY ,  "Undefined" ) )  AND  ( LIMIT-TO ( DOCTYPE ,  "ar" ) )  AND  ( LIMIT-TO ( SUBJAREA ,  "MEDI" ) )  AND  ( LIMIT-TO ( PUBYEAR ,  2019 )  OR  LIMIT-TO ( PUBYEAR ,  2018)  OR  LIMIT-TO ( PUBYEAR ,  2017 )  OR  LIMIT-TO ( PUBYEAR ,  2016 )  OR  LIMIT-TO ( PUBYEAR ,  2015 )  OR  LIMIT-TO ( PUBYEAR ,  2014 )  OR  LIMIT-TO ( PUBYEAR ,  2013 )  OR  LIMIT-TO ( PUBYEAR ,  2012 )  OR  LIMIT-TO ( PUBYEAR ,  2011 )  OR  LIMIT-TO ( PUBYEAR ,  2010 )  OR  LIMIT-TO ( PUBYEAR ,  2009 )  OR  LIMIT-TO ( PUBYEAR ,  2008 )  OR  LIMIT-TO ( PUBYEAR ,  2007 )  OR  LIMIT-TO ( PUBYEAR ,  2006 )  OR  LIMIT-TO ( PUBYEAR ,  2005 )  OR  LIMIT-TO ( PUBYEAR ,  2004 )  OR  LIMIT-TO ( PUBYEAR ,  2003 )  OR  LIMIT-TO ( PUBYEAR ,  2002 )  OR  LIMIT-TO ( PUBYEAR ,  2001 )  OR  LIMIT-TO ( PUBYEAR ,  2000 ) )  AND  ( LIMIT-TO ( LANGUAGE ,  "English" )  OR  LIMIT-TO ( LANGUAGE ,  "Italian" ) )  AND  ( LIMIT-TO ( EXACTKEYWORD ,  "Human" ))

**Web of Science**

(TS=(("secondary care" OR "tertiary care" OR hospital) AND ("biomedical technology"  OR "technology")  AND  (organizational  OR  management  OR  barrier  OR  facilitator  OR  enabler)  AND  (adoption  OR  implementation  OR  acceptance  OR  evaluation  OR  assessment  OR  measurement  OR  utilization  OR  assimilation )))  AND DOCUMENT TYPES: (Article)
Refined by: LANGUAGES: ( ENGLISH OR ITALIAN ) AND RESEARCH AREAS: ( HEALTH CARE SCIENCES SERVICES OR MEDICAL INFORMATICS OR COMPUTER SCIENCE OR OPHTHALMOLOGY OR GENERAL INTERNAL MEDICINE OR PUBLIC ENVIRONMENTAL OCCUPATIONAL HEALTH OR GASTROENTEROLOGY HEPATOLOGY OR INFORMATION SCIENCE LIBRARY SCIENCE OR SURGERY OR HEMATOLOGY OR MEDICAL LABORATORY TECHNOLOGY OR ENGINEERING OR REPRODUCTIVE BIOLOGY OR PHARMACOLOGY PHARMACY OR CARDIOVASCULAR SYSTEM CARDIOLOGY OR PEDIATRICS OR IMMUNOLOGY OR MICROBIOLOGY OR RADIOLOGY NUCLEAR MEDICINE MEDICAL IMAGING OR NEUROSCIENCES NEUROLOGY OR ONCOLOGY OR OBSTETRICS GYNECOLOGY OR INFECTIOUS DISEASES OR RESPIRATORY SYSTEM OR SCIENCE TECHNOLOGY OTHER TOPICS OR TELECOMMUNICATIONS OR UROLOGY NEPHROLOGY OR BIOTECHNOLOGY APPLIED MICROBIOLOGY OR INSTRUMENTS INSTRUMENTATION ) AND WEB OF SCIENCE INDEX: ( WOS.SCI ) AND DOCUMENT TYPES: ( ARTICLE )
Timespan: 2000-2019. Indexes: SCI-EXPANDED, SSCI, A&HCI, CPCI-S, CPCI-SSH, BKCI-S, BKCI-SSH, ESCI, CCR-EXPANDED, IC.

**OVID MEDLINE**

(("secondary care" or "tertiary care" or hospital) and ("biomedical technology" or "technology") and (organizational or management or barrier or facilitator or enabler) and (adoption or implementation or acceptance or evaluation or assessment or measurement or utilization or assimilation)).ti,ab.

1 Risorsa selezionata/e : Ovid MEDLINE(R) ALL

limit 1 to yr="2000 - 2019"
limit 2 to (english or italian)

Termini di ricerca utilizzati:

- acceptance
- adoption
- assessment
- assimilation
- barrier
- biomedical
- technology
- enabler
- evaluation
- facilitator
- hospital
- implementation
- management
- measurement
- organizational
- secondary
- care
- tertiary
- utilization

**ECONLIT**
(("secondary care" OR "tertiary care" OR  hospital)  AND  ("biomedical technology"  OR "technology")  AND  (organizational  OR  management  OR  barrier  OR  facilitator  OR  enabler)  AND  (adoption  OR  implementation  OR  acceptance  OR  evaluation  OR  assessment  OR  measurement  OR  utilization  OR  assimilation))

 Riviste accademiche

 2000-01-01 - 2019-01-01

 Articolo

 NOT (mortality AND economics of gender, non-labor discrimination (j16) AND environmental economics: government policy (q58) AND game theoretic AND game theory and bargaining theory: general (c70) AND games AND gender AND morbidity)

 Inglese
