# Supplementary material for: Hospital contextual factors affecting the implementation of health technologies: a systematic review
Source: BMC Health Serv Res. 2021 May 1;21:407. doi: 10.1186/s12913-021-06423-2 (PMC8088675; doi:10.1186/s12913-021-06423-2)
Supplement: Supplementary file 5 — Additional file 5. [file 12913_2021_6423_MOESM5_ESM.docx]

**Title:** Hospital contextual factors affecting the implementation of health technologies: A systematic review

**Authors**:
Grossi Adriano^1^*, Hoxhaj Ilda^1^*, Gabutti Irene^2^, Specchia Maria Lucia^1^, Cicchetti Americo^2^, Boccia Stefania^1,3^, de Waure Chiara^4^

**Affliations:**1. Section of Hygiene, Univesity Department of Life Sciences and Public Health, Università Cattolica del Sacro Cuore, Roma, Italia
2. ALTEMS – Faculty of Economics, Università Cattolica del Sacro Cuore, Rome, Italy
3. Department of Woman and Child Health and Public Health - Public Health Area, Fondazione Policlinico Universitario A.Gemelli IRCCS, Roma, Italia
4. Department of Experimental Medicine, University of Perugia, Perugia, Italy

*Equal contribution

**Additional file 5.** Quality assessment of three included studies in the systematic review using Mixed Methods Appraising Tool.

| **First author, Year** | **Is there an adequate rationale for using a mixed methods design to address the research question?** | **Are the different components of the study effectively integrated to answer the research question?** | **Are the outputs of the integration of qualitative and quantitative components adequately interpreted?** | **Are divergences and inconsistencies between quantitative and qualitative results adequately addressed?** | **Do the different components of the study adhere to the quality criteria of each tradition of the methods involved?** |
| --- | --- | --- | --- | --- | --- |
| Vadillo | yes | no | no | no | yes |
| Edmonson | yes | yes | yes | yes | yes |
| Moeckli | yes | yes | yes | yes | yes |
