# Supplementary material for: Hospital contextual factors affecting the implementation of health technologies: a systematic review
Source: BMC Health Serv Res. 2021 May 1;21:407. doi: 10.1186/s12913-021-06423-2 (PMC8088675; doi:10.1186/s12913-021-06423-2)
Supplement: Supplementary file 6 — Additional file 6. [file 12913_2021_6423_MOESM6_ESM.docx]

**Title:** Hospital contextual factors affecting the implementation of health technologies: A systematic review

**Authors**:
Grossi Adriano^1^*, Hoxhaj Ilda^1^*, Gabutti Irene^2^, Specchia Maria Lucia^1^, Cicchetti Americo^2^, Boccia Stefania^1,3^, de Waure Chiara^4^

**Affliations:**1. Section of Hygiene, Univesity Department of Life Sciences and Public Health, Università Cattolica del Sacro Cuore, Roma, Italia
2. ALTEMS – Faculty of Economics, Università Cattolica del Sacro Cuore, Rome, Italy
3. Department of Woman and Child Health and Public Health - Public Health Area, Fondazione Policlinico Universitario A.Gemelli IRCCS, Roma, Italia
4. Department of Experimental Medicine, University of Perugia, Perugia, Italy

*Equal contribution

**Additional file 2.** Main findings related to organizational barriers

| **First Author** | **Year** | **Main ogranizational factors** |
| --- | --- | --- |
| Bomba D. | 2006 | - If EPDS required more time to prescribe it would create a serious issue  - Training and support was highlighted as a concern by both clinicians and experts, outlining the difficulty in providing training in a 24-hour  - Ease of use was  related to minimal keystrokes and ensuring simple navigation  - Lack of funding was a commonly cited reason for lack of adoption  - Lack of computer hardware on wards and the current state of disparate clinical IT systems |
| Dharampl N. | 2016 | - The SSC was difficult to implement owing to a shift in time management and perioperative culture.  - The utilization of the SSC was enabled by ubiquitous awareness, perceived relative advantage and compatibility with existing ideals.  - The barriers of adoption include complexity of integration into the current perioperative workflow, difficult trialability and lack of observability of the desired effect. |
| Bonada N.S. | 2016 | Difficulties in implementation:  - Lack of interoperability and integration between systems  - Difficulty to manage clinical information  - Constant maintenance and update of information, with changes required according to the use and assessment of alerts  - The high cost of implementation and maintenance |
| Cucciniello M. | 2015 | - Extensive training courses arranged at the initial phase to guide the introduction of the new system  - Defining and resourcing new roles within the organization capable of supporting and sustaining the change  - Staff involvement and the level of commitment was independent of the adoption of the EMR system |
| Debono D. | 2017 | Barriers to EMMS use:  - Environmental Context and Resources  - Not impinging on their colleagues’ time   -  The perception that doing so would impede providing patient-centred care  - Time pressure and competing demands  - Unavailability of COWs when taking the eMAR to a patient when administering medication |
| Duyck P. | 2010 | - The availability of support (education and training through a project team) is more important when users are alredy using the technology.  - The impact and usefulness of the technoogy affect the scale of use |
| Edmondson A. C. | 2003 | Facilitators were:  - Team leader coaching  - Communication (ease of speaking up)  - Boundary spanning (actions neede to coordinate objectves, schedules and rousources with those of the organization) |
| Granlien M.F. | 2008 | Barriers to EMR implementation: 1. related to human factors:  - Lack of knowledge, information, and training  - Uncertainty about what constitutes the barriers to using different parts of the EMR 2. related to system factors:   - Poor usability and overview  - Inadequate support of certain work areas  - Time: the system being too slow and time consuming to use |
| Hao H. | 2011 | - Opinion leader influence their peers’ adoption behavior |
| Hubner U | 2010 | - Good-infrastructure of medical-technical devices  - Rigourous organizational changes which have led to leaner processes and to a lower lenght of stay and a more friendly IT climate |
| Kazley AS | 2007 | At the multivariate model are: larger hospitals and not rural. |
| Merkel S. | 2015 | Detereminants to TAVI implementation:  - Reimbursement policies and cost issues  - The collaboration between cardiologists and cardiac surgeons, negatively affected the implementation and difussion  - Champions, eager to implement the method, facilitated the dialogue between both departments and took initiative to drive the process forward  - Organizational requirements needed to implement the procedure, such as the heart team |
| Nakamura M.M. | 2013 | Hospital teaching status is related to EHR adoption |
| Moeckli J. | 2013 | Organizational barrier post-implementation:  - Understanding and expectations: staff  did not know how and when to use it  - Impact on work systems and perceived usefulness |
| Nanji K.C. | 2009 | - Process flow issues due to the need to redisegn workflow  - Need of long-term on-site formal training and support and  to maximize workflow customization with specific changes to its system  - Clear communication around workload expectations during the implementation process may mitigate misunderstandings and the resulting staff resistance.  - Changing roles were also an important cause of resistance among staff.  - Champions could be facilitators  - The bar code scanning technology also facilitated collaboration and teamwork, which served as a catalyst for system adoption. |
| Olson J.R. | 2012 | -Degree of implementation of IT depends on technology capability, which is based on technological context and organizational context. |
| Paré G | 2007 | - Active and sustainded involvement of highly motivated actors with complementary skills and interests is likely to favour project success  - Key actors must exert enough decision-making power to oversee the PACS acquisition process e.g. radiologist by professional bureaucracies  - Economic barrier fell when it could be demonstrate that the project cover its own costs  - Group of influencial actors and proof of financial self-sufficiency represent necessary conditions for project success, but they in themselves are not sufficient  - Do not beleive in "magic thinking" rather adopt proactive strategy that take into account several aspects. For example:  In Hospital alfa, successful actions were: a reorganization of the imaging department, seeking voluntary departures, adjusting to processes and tasks over a 10-12 week period, strong communication lasting up to post-acquisition phase. Hospital beta focused essentially on technical aspects and not on organizational and human factors resulting in less favourable output. |
| Randell R. | 2010 | - Financial difficulties in recruiting and retaining technical staff  - For nurses, engagement of clinicians in the procurement and implementation is useful  - Engaging nurses with the process of introducing CDSS can facilitate succesfull use of system but it might not be necessary  - Formal mechanisms for IT procurement: ensuring the technology can be adapted to the needs of the working environment and recognise that implementation is not just a "technical"project  - Education: training was difficult to organize because nurses have difficulty being released form clinical area  - Organizational culture: supportive nature of the environment to innovate and improve practice and the desire to improve quality of the assistance |
| Pare G. | 2010 | - Hospital size resulted one of the strongest predictors of HIT innovativeness  - Hospitals in urban locations showed a higher level of HIT innovativeness |
| Poon E.G. | 2004 | - Strong leadership was a recurring theme in almost all interviews. Hospital leaders had to be firm believers to demonstrate visibly a commitment to the implementation project. They need to be facile at managing changes that inevtibaly came.Some managers led by example and wer among the fist to adopt it.   - Identifying physician champions: were key to succesfull CPOE implementation. During the rollout, cahmpins would remind the general users of its downstram benefits, encoruraging them to see beyoind their immediate frustrations. They would also relay users'concerns to the implementation team and the vendors  - Addressing workflow conerns: the frustration thar came with learning to use anew ordering system often was mitigated by the presence of support staff.  - Leveraging house staff or hospitalists:facilitators were considered hospitalist and medicine students that were already been exposed to the technoloy strong and objective buisness case for CPOE  - Some hospital mangers believed that financial incentives could come from either the government or private insurace companies.  - Improve system interoperability |
| Scholten N. | 2015 | - Hospital size (500 to 1000 beds compared to smaller and larger hospitals) had a negative influence on implementation  - Stroke unit have a positive influence on implementation  - Teaching status, location, ownership and number of treated ischemic srtroke patients did not influence the higher implementation during the study period |
| Sheikh A. | 2011 | - top-down national strategy rushed to the implementation, sometimes with adverse consequences, such as considerable time and efforts to work our new technolgy, need of training and support, higher workload for clinicians, unrealistic expectations from clinicians |
| Shen X. | 2012 | Barriers to EHR implementation:  - Unexpected difficulties in implementation  - Inadequate support services  - Cost of implementation  - Difficult to integrate with hospital system  - Insufficient human resources  - Lack of guideline for radiation oncology |
| Sommerbakk R. | 2016 | Barrier to implementation:  - Lack of leadership (e.g. distant management, lack of support, nurses not represented in leadership group)  - Lack of support from colleagues  - Low human resources  - Lack of time  - Lack of expertise of QI nurse  - Lack of training  - Lack of extra funding |
| Struik M.H | 2014 | - A performance feedback system can make clinicians aware of their delivered quality of care  - Respondents did not prefer to get training for the EMR  - For nurses, the stimulating attitude by the head of department  - For physicians, decision support  - To get support of IT helpdesk |
| Szydlowski S. | 2009 | Barriers to HIT implementation:  - Not properly  managing expectations of HIT (CIO)  - Time-consuming trainings (CIO and nurse managers)  - Resource shortage (CIO)  - Poor communication and leadership (nurse managers)  - Need of IT education (CIO and nurse managers)  - Staff shortages (nurse managers) |
| Takian A. | 2014 | - Omega hospital, compared to Alfa hospital, learned to get engaged with users from an early stage and encouraged learning through user-friendly and innovative tailored training  - In addition, crucial was an experienced and insightful leadership and managerial team who were signed-up to the vision of EHR, had greater ownership of the implementation than Alpha and acted as boundary spanner that bridged the gap between the implementation team and end users |
| Tuot D.S. | 2015 | Facilitators were:  - executive and clinician leadership  - early clinician adopters  - commitment to optmize clinician workflow  - funding model for specialist clinician reimbursement Barriers were:  - lack of integration with EHR  - change in workflow  - lack of reimbursement mechanisms |
| Urowitz S | 2008 | - Hospital financial resources the most important barrier to implementation |
| Vadillo P.C. | 2016 | - Importance of providing proper training in basic computer functions  - Importance of having an IT support person, such as an appointed "super-user", on site durgin the first weeks of implementation |
| Varonen H. | 2008 | Barriers to CDSS implementation:  - Earlier experience of dysfunctional computer systems in health care  - General resistance towards changes in practice  - Time management issues  - Issues of compatibility and updating, problems with several poorly interacting computer programmes  - Obscured responsibilities; loss of own reasoning and clinical autonomy Facilitating factors:  -  Flexibility of the system; possibility to tailor the selection of topics or patients for reminders and possibility to switch of the system  -  Reliability; reliable knowledge base and that trusted peers are developing the system  -  Simplicity and ease of use  - Adequate budgeting  - Concise reminders that facilitate and help work processes  - Concise and tailored education for the use of CDSS |
| Woiceshyn J. | 2017 | 1. The integrated innovation mode manifests in the leaders' framing and communicating innovation initiatives as opportunities. 2. Executive champion:   - The leaders seek to integrate them with existing projects so that they make sense to the staff.  - The leaders support the frontline staff, for example, by inviting and incorporating staff's input in the implementation, providing a clear vision for integrating initiatives, training, and additional support 3. Lacking implementation resources. Although resources were scarce also at the effective implementation sites, the work-in-progress sites felt the resource shortage more acutely and were less able to make trade-offs. |
| Xie Y. | 2016 | Enablers: - organization size  - top management support  - the availability of sufficient resources Barrier:  - lack of resources |
